# Quantifying Chemotherapy Delivery in Older and Younger Women With Early-Stage Breast Cancer Using Longitudinal Cumulative Dose

**DOI:** 10.1093/oncolo/oyad349

**Published:** 2024-02-08

**Authors:** Joyce Pak, Kirsten A Nyrop, Hyman B Muss, Moriah K Forster, Jennifer L Lund

**Affiliations:** Department of Epidemiology, University of North Carolina at Chapel Hill, Chapel Hill, NC, USA; Lineberger Comprehensive Cancer Center, University of North Carolina, Chapel Hill, NC, USA; Department of Medicine, School of Medicine, University of North Carolina, Chapel Hill, NC, USA; Lineberger Comprehensive Cancer Center, University of North Carolina, Chapel Hill, NC, USA; Department of Medicine, School of Medicine, University of North Carolina, Chapel Hill, NC, USA; Division of Hematology and Oncology, Department of Medicine, Vanderbilt University, Nashville, TN, USA; Department of Epidemiology, University of North Carolina at Chapel Hill, Chapel Hill, NC, USA

**Keywords:** breast cancer, chemotherapy, longitudinal cumulative dose

## Abstract

**Background:**

Delivery of cancer treatments, such as chemotherapy, requires a complex set of decisions that can change over time. Traditional measures of chemotherapy delivery, such as relative dose intensity, measure the amount of chemotherapy received by the end of treatment but mask the timing of dose reductions, delays, and discontinuation. These events may be important for delivering timely interventions to support adherence and lower the risk of recurrence.

**Materials and Methods:**

We used an institutional database to identify women diagnosed with stage I-III breast cancer receiving adjuvant chemotherapy with a standard 4-cycle regimen of docetaxel + cyclophosphamide (TC, every 21 days) from April 2014 to December 2019. LCD was calculated as the amount of a given chemotherapy agent delivered at a specified time, *t*, divided by the total planned standard chemotherapy dose at time *t*. We visualized LCD curves for each chemotherapy agent and reported the median LCD and interquartile range (IQR) at the end of the regimen, overall and by age group (<65 years vs. 65+ years).

**Results:**

The study population included 80 women. At the end of treatment, overall median LCDs for both cyclophosphamide and docetaxel were 100% (IQR: 99.6%, 100%), suggesting that TC was well tolerated. However, the lower quartile LCD for cyclophosphamide was 98.7% in older women treated with TC compared with 99.7% in younger women.

**Conclusion:**

Within our cohort, adjuvant TC was well tolerated with LCD curves showing largely on-time and full-dose administration. Subgroup analyses showed only slight decreases in adjuvant TC LCD for patients aged 65+ versus <65 years.

## Teaser

The longitudinal cumulative dose (LCD) describes chemotherapy delivery by cycle and integrates any information about delays, reductions, missed doses, or early discontinuation of therapy into one continuous measure. This article describes chemotherapy delivery among women with early breast cancer using the novel LCD measure overall and by age group.

**Implications for Practice**

The longitudinal cumulative dose describes chemotherapy delivery by cycle and integrates any information about delays, reductions, missed doses, or early discontinuation of therapy into one continuous measure. We can move away from summarizing chemotherapy dosing at the end of treatment to understand the dynamics of treatment patterns over the entire course of therapy, which will be important for delivering timely interventions to support adherence and potentially lower the risk of recurrence.

## Introduction

Delivery of cancer treatments such as chemotherapy requires a complex set of decisions on the part of patients and healthcare providers that can change within the treatment period. For example, after the start of a patient’s chemotherapy treatment, patients may experience toxicity that causes treatment delays, dose reductions, missed doses, or early treatment discontinuation. The occurrence of these events varies by patient, drug, and regimen.^1^ Traditional measures of chemotherapy delivery, such as relative dose intensity (RDI), measure the total amount of chemotherapy received by the end of treatment as compared to the planned regimen, but do not provide information about the timing of changes in the treatment plan.^1,2^ These events may be important for delivering timely interventions to support adherence and lower the risk of recurrence.

A new measure, the longitudinal cumulative dose (LCD), can describe chemotherapy delivery for an individual over time by integrating multiple dimensions of treatment (ie, delays, reductions, and stopping treatment) into one simple metric. LCD can then be aggregated and used to quantify patterns of treatment delivery across patient subgroups.^1^ The LCD has been previously used to examine oxaliplatin and fluorouracil delivery in patients with colon cancer in both trial and clinical practice settings.^1,3^ In the trial setting, agreement between the LCD and RDI was 0.39, indicating only fair agreement between the two measures.^1,3^ The observed differences between the LCD and RDI were primarily driven by high levels of treatment modifications over time. Considering that treatment for early-stage breast cancer (EBC) is generally well-tolerated, we explore the LCD in the context of treatment for EBC. Patients diagnosed with EBC who require dose reductions and treatment delays are more likely to experience disease recurrence and death compared to those who complete their full chemotherapy dose on time.^4^ Further, compared to younger women, older women and those who are frail with a diagnosis of EBC experience increased chemotoxicity, resulting in dose reductions and treatment delays.^4,5^ To this end, we aimed to describe chemotherapy delivery among women with EBC treated in a single institutional database using the novel LCD measure overall and by age group.

## Materials and Methods

### Data Source and Study Population

We used an existing university-affiliated hospital center database collected from previously published studies.^6,7^ Our dataset consisted of 725 female patients, aged 18 or older, diagnosed with stage I-III breast cancer receiving chemotherapy, all of whom did not have evidence of breast cancer recurrence, progression, or metastasis within 2 years post-primary treatment.^6,7^ All patients had detailed treatment information abstracted from the electronic health record, including weight data from breast cancer diagnosis through 2 years post-primary treatment.

For the current analysis, we restricted our study sample to patients diagnosed between April 2014 and December 2019 who initiated a 4-cycle regimen of docetaxel/cyclophosphamide (TC, every 21 days). TC was intentionally selected for this study because it is among the most commonly used adjuvant chemotherapy regimens for early breast cancers and the preferred adjuvant chemotherapy of choice for older women.^8,9^ All treatment data were accessed via Research Electronic Data Capture database hosted by the University of North Carolina (UNC) School of Medicine. The study was approved by the UNC’s Institutional Review Board (IRB #: 12-0045).

### Relative Dose Intensity

The RDI, a summary statistic used to assess the total dose of chemotherapy received, is defined as the ratio of the delivered dose intensity to the planned dose intensity at the end of treatment.^2,10^ For example, for 4-cycle chemotherapy agents infused every 21 days, a patient with no dose reductions or delays has an RDI of 100% on day 64. If treatment was stopped by the provider after the third cycle, but until that point, the patient received full and on-time treatment, their RDI should be 75% which would account for the expected number of days for the final missed cycle.^2^

### Longitudinal Cumulative Dose

The LCD was calculated to quantify chemotherapy delivery for each patient by integrating the impacts of dose reductions, missed doses, and dose delays over time.^1^ LCD is defined as the amount of a specific chemotherapy agent delivered at a specified time, *t*, divided by the total planned standard chemotherapy dose, with both the numerator and denominator adjusting for body surface area.^1^ A delay was defined as chemotherapy given 3 or more days later than indicated in the initial treatment plan. For example, for 4-cycle chemotherapy agents infused every 21 days, a patient with no dose reductions or delays has an LCD of 25% on days 1-21, 50% on days 22-42, 75% on days 43-63, and reaches an LCD of 100% on day 64. If the patient experiences a dose reduction by half after receipt of the initial dose, they have an LCD of 25% from days 0 to 21, and after 4 cycles, their LCD will be 62.5%. Like the RDI, the LCD at the end of treatment can also be over 100% if a patient received extra infusions or higher than standard doses. Individual-level LCDs can then be aggregated to quantify population-level LCD summary statistics that can be compared across subgroups.^3^ If calculated appropriately, the LCD and the RDI should be equivalent at the end of planned treatment.

### Statistical Analyses

Descriptive statistics were used to summarize the study participant’s demographic and clinical characteristics. For the overall population, we visualized LCD curves for each chemotherapy agent (cyclophosphamide and docetaxel) in patients receiving adjuvant TC and reported the median LCD and interquartile range (IQR) at the end of the regimen. We also explored LCD curves stratified by age groups (<65 years vs. ≥65 years). All analyses were conducted in SAS version 9.4 (SAS Institute, Cary, NC).

## Results

Table 1 presents the characteristics of 80 patients with EBC diagnosis who received adjuvant TC chemotherapy. Among the patients who received TC chemotherapy (*n* = 87), the majority of patients received adjuvant TC (*n* = 80) compared to neoadjuvant TC (*n* = 7). Among the adjuvant TC patients, the mean (SD) age at EBC diagnosis was 58.3 years (11.3) and 43.8% were ≥65 years old. A total of 61 patients (76.3%) self-identified as White and 14 (17.5%) self-identified as Black. More than 80% of patients initiating adjuvant TC completed the 4 cycles of chemotherapy. At the end of treatment, overall median RDIs for cyclophosphamide and docetaxel were 100% (IQR: 99.6%, 100%) and 100% (IQR: 98.4%, 100%), respectively.

**Table 1.** Selected baseline and treatment-related characteristics of patients by adjuvant and neoadjuvant TC.

Characteristic, n (%)	Adjuvant TC (n = 80)	
	No.	%
Age at BC diagnosis		
Under age 65	45	56
65 and older	35	44
Age at BC diagnosis (mean, sd, range)		
Total	58.33 (11)	(24, 77)
Under age 65	50.78 (9.4)	(24, 64)
65 and older	68.03 (3.3)	(65, 77)
Race		
White	61	76
Black	14	18
Other	5	6
Stage		
I	34	43
II	43	54
III	3	4
ER+	59	74
PR+	53	66
HER2+	4	5
Radiation	58	73
Completed 4 study cycles	66	83
Final LCD for cyclophosphamide (median, P25-P75)	100% (99.6%-100%)	
Final LCD for docetaxel (median, P25-P75)	100% (98.4%-100%)	

Abbreviations: LCD, longitudinal cumulative dose; P25, 25th percentile; P75, 75th percentile; TC, docetaxel/cyclophosphamide.

**Figure 1.**
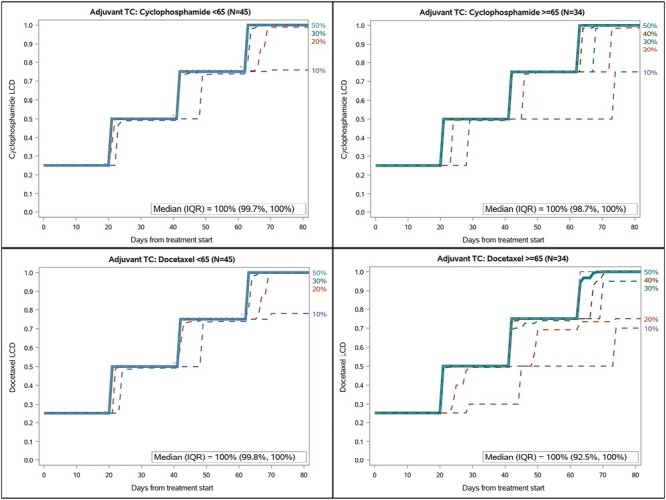
LCD plots for the EBC analyses.

The combined impacts of dose reductions, delays, and discontinuations can be seen in Figure 1, which includes 4 plots of the LCD over time, each corresponding to a different regimen (cyclophosphamide and docetaxel) stratified by age group (<65 and ≥65 years). Each panel presents the 10th through 90th percentiles for their respective agent’s cumulative dose across 72 days following chemotherapy initiation. Overall, TC was well tolerated, with only slight delays observed between the third and fourth cycles. At the end of treatment, overall median LCDs for cyclophosphamide and docetaxel were 100% (IQR: 99.6%, 100%) and 100% (IQR: 98.4%, 100%), respectively. However, in older women, the lower quartile LCD for cyclophosphamide was 98.7% compared with 99.7% in younger women. The difference in lower quartile LCD for docetaxel was more pronounced with 92.5% in older women compared to 99.8% in younger women.

## Discussion

The docetaxel/cyclophosphamide regimen is among the most commonly used and effective adjuvant treatments for breast cancer.^11,12^ In this institutional-based cohort, the LCD showed that adjuvant TC was well-tolerated showing largely on-time and full-dose administration. Differences when comparing final LCD with RDI were negligible further suggesting that patients were adhering to their ideal treatment course. This finding supports previous studies, as dose delays and reductions are least frequently observed in the TC regimen.^13^ However, if a patient is not adhering to their ideal treatment course, the RDI will overestimate treatment adherence as RDI can be misleading when patients stop their treatment early and the missed days of treatment are not included. While LCD steadily increments over time (ie, patients reach a final LCD of 100% after receiving the full standard dose of a given agent), the RDI is a fixed measure once a patient stops their treatment. Thus, examining chemotherapy patterns using a cycle-specific reporting of treatment adherence (eg, the LCD) provides a clearer understanding of the chemotherapy actually received by patients over time.

When stratified by age and regimen, the study demonstrated that older women receiving adjuvant TC lagged on-time and full-dose administration with differences emerging after 48 days, a finding that would be masked by the RDI. This finding is consistent with previous studies, as patients with EBC ≥65 years had suboptimal RDI tolerance of adjuvant regimens and were more likely to have dose delays and reductions.^5,13,14^ However, a growing body of evidence suggests that older patients can tolerate a range of adjuvant regimens better than previously thought and are capable of maintaining optimal dose intensity.^4,5^ This can be attributed to the heterogeneity in aging as aging is a highly complex and multifaced process. The difference in lagged on-time and full-dose administration was more pronounced in older adults receiving taxane-based adjuvant chemotherapy regimens. Data with taxane-based adjuvant chemotherapy regimens in the older population remain very limited,^4^ and our finding shows that even at the lowest quartile, adjuvant taxane-based TC is still well-tolerated at 92.5%. Although the clinical effect of LCD differences is unknown, future analyses could evaluate patient characteristics associated with lag and low LCD and potentially target those populations for further supportive care or navigation interventions.

### Limitations

There are limitations of this study that should be considered. First, this is a single-institution study at a university-affiliated hospital; as such, the results may fail to generalize to other populations, particularly to populations treated in community practices. Of note, however, these patients were not part of a clinical trial and are likely to be representative of patients with breast cancer receiving adjuvant therapy in the general population. Second, all results need to be interpreted cautiously, considering the descriptive nature of our study objectives and the inherent limitation of small sample sizes. Because of these limitations, we refrained from conducting any statistical analyses or additional stratification. Third, our work is a secondary analysis of data collected from previously published studies,^6,7^ that excluded patients with EBC who experienced recurrence in the 2 years following diagnosis. This exclusion in the underlying dataset may have led to higher LCD estimates than if those patients had been included. Lastly, while our work was helpful for understanding chemotherapy treatment delivery, we were limited to estimate quantitative differences in LCD. Future work could explore associations between different levels of LCD and clinical (relapse-free survival) and patient-centered outcomes (ie, cognition, quality of life, and patients’ frailty) could yield additional insights that may inform patient care. Further, this metric can be used in studying more toxic regiments and their association with recurrence and survival outcomes.

## Conclusion

The LCD describes chemotherapy delivery by cycle and integrates any information about delays, reductions, missed doses, or early discontinuation of therapy into one continuous measure. Using this novel metric, we demonstrated that within our cohort of women with stage I-III breast cancer, adjuvant TC was well-tolerated showing largely on-time and full-dose administration. Subgroup analyses showed only slight decreases in the LCD for adjuvant TC among patients aged 65+ versus <65 years.

## Data Availability

The data underlying this article were provided by a university-affiliated hospital center under the UNC’s Institutional Review Board (IRB #: 12-0045). Data will be shared on request to the corresponding author with permission of UNC.
